# Commercial weight loss diets meet nutrient requirements in free living adults over 8 weeks: A randomised controlled weight loss trial

**DOI:** 10.1186/1475-2891-7-25

**Published:** 2008-09-02

**Authors:** Helen Truby, Rebecca Hiscutt, Anne M Herriot, Manana Stanley, Anne deLooy, Kenneth R Fox, Susan Baic, Paula J Robson, Ian Macdonald, Moira A Taylor, Robert Ware, Catherine Logan, MBE Livingstone

**Affiliations:** 1 Children's Nutrition Research Centre, Royal Children's Hospital, Herston, Qld, Australia, 4029; 2 Faculty of Health Sciences, University of Surrey, Guildford, GU2 7XH, UK; 3 Faculty of Health Sciences, University of Plymouth, Plymouth, PL6 9BH, UK; 4 Department of Exercise and Health Sciences, University of Bristol, Bristol, BS8 1TP, UK; 5 Northern Ireland Centre for Food and Health, University of Ulster, Coleraine, BT52 1SA, Northern Ireland; 6 School of Biomedical Sciences, University of Nottingham, Nottingham, NG7 2UH, UK; 7 School of Population Health, University of Queensland, Herston, Australia, 4029

## Abstract

**Objective:**

To investigate the effect of commercial weight loss programmes on macronutrient composition and micronutrient adequacy over a 2 month period.

**Design:**

Adults were randomly allocated to follow the Slim Fast Plan, Weight Watchers Pure Points Programme, Dr Atkins' New Diet Revolution, or Rosemary Conley's "Eat Yourself Slim" Diet & Fitness Plan.

**Setting:**

A multi-centre randomised controlled trial.

**Subjects:**

293 adults, mean age 40.3 years and a mean BMI 31.7 (range 27–38) were allocated to follow one of the four diets or control group. Subjects completed a 7-day food and activity diary at baseline (prior to randomisation) and after 2 months. Diet records were analysed for nutrient composition using WinDiets (research version).

**Results:**

A significant shift in the macronutrient composition of the diet with concurrent alteration of the micronutrient profile was apparent with all diets. There was no evidence to suggest micronutrient deficiency in subjects on any of the dietary regimens. However, those sub-groups with higher needs for specific micronutrients, such as folate, iron or calcium may benefit from tailored dietary advice.

**Conclusion:**

The diets tested all resulted in considerable macronutrient change and resulted in an energy deficit indicating dietary compliance. Health professionals and those working in community and public health should be reassured of the nutritional adequacy of the diets tested.

**Trial Registration Number:**

NCT00327821

## Introduction

The slimming industry is a thriving and lucrative business in many developed countries throughout the world. In the UK, at any one time, it is estimated that almost two in five women and one in six men are on some kind of reducing diet. The continued rise in obesity supported by the obesogenic environment, combined with a desired society image of a 'slim body' being ideal [1] suggests that this situation is likely to continue. There is a plethora of weight loss regimens marketed at the lay public. The theories underpinning these, and the marketing techniques used to sell them range from little scientific basis to having a reasonably robust scientific basis [2]. The popularity of a given diet often bears little relation to the scientific evidence base for its efficacy [3].

An excellent example of the impact and resulting confusion that a diet can cause among both the lay public, academics and health professionals is the internationally popular low carbohydrate (CHO) Atkins diet [4]. The potential detrimental health effects such a dramatic alteration in macronutrient intake could provoke has caused concern in many esteemed professional bodies [5]. The current lack of credible evidence about its long term safety has meant that health practitioners have been divided in their response [6] with most dismissing it as a viable or safe weight loss regimen. However, there is a growing evidence base that suggests reduced CHO diets may not have all the adverse effects on cardiovascular risk factors and especially lipid profile previously postulated [7-9]. In short term studies (up to 12 months), low CHO diets seem to be at least as effective in achieving weight loss as more widely endorsed methods of energy reduction, such as low fat diets [8,7,10,11]. However, there is no information in the literature to date that reports extensively on the actual nutritional composition of a low CHO diet in free-living subjects.

The scale of the obesity problem and the limitations within health systems to provide weight management advice, mean that the vast majority of people trying to lose weight are likely to do so on their own initiative and use whatever sources of information they have to hand. Thus evaluation of the effectiveness and impact of popular diets is important and has not to date been rigorously investigated. There is very little information on the effect that commercial diets have on the food choices of people undertaking these regimens in an unsupervised free-living population. In particular, there may be implications for the micronutrient sufficiency for adults following energy restricted diets, especially for those following low carbohydrate approaches.

'Diet Trials', a large UK multi-site randomised controlled study was designed to compare the relative efficacy of four commercial weight loss programmes on weight and body fat loss and the primary outcomes of this study have recently been published [11]. The diets were chosen as being representative of the major different approaches to weight management available in the UK: the Slim-Fast Plan (a meal replacement approach), Weight Watchers Pure Points Programme (an energy controlled diet with weekly group meetings), Dr Atkins' New Diet Revolution (a low carbohydrate eating plan) and Rosemary Conley's "Eat Yourself Slim" Diet & Fitness Plan (a low fat diet combined with a weekly group exercise class). A time period of 8 weeks was chosen to measure changes as this initial period is often the greatest phase of compliance and when the majority of weight changes are demonstrable.

In addition to investigating the effectiveness in achieving weight loss, this study provided an evaluation of the degree to which subjects following commercial weight loss programmes with no additional assistance and self-selecting foods were able to make dietary choices that were consistent with the advice provided by the commercial organisations. Another novel aspect of this study was to investigate if any of these programmes compromised micronutrient intakes during a two month period of energy reduction. The nutritional composition of a low CHO diet was compared to the low fat diets (WW and RC). This aspect of the study addresses a shortfall in the literature and will provide health professionals and other bodies with evidence about the nutritional adequacy of popular diets.

The aim of this paper is thus to report the dietary macro- and micronutrient changes that occurred in the first two months of dieting in a group of overweight adults taking part in 'Diet Trials'.

## Method

Ethical approval for the study was obtained from the South East Multi-centre Research Ethics Committee.

### Subject recruitment and randomisation

Three hundred subjects were recruited via media advertising across the UK. Subjects were assigned to attend one of the five regional centres (60 at each centre), situated at the Universities of Surrey, Bristol, Nottingham, Ulster (Coleraine) and Queen Margaret University College, Edinburgh. Standardised assessment instruments and protocols were distributed by the lead centre prior to the study commencing. Participants were considered eligible if they fulfilled the following criteria: aged 18–65 years, had a BMI > 27 and < 40 kgm^2^, were not actively dieting and lived within a 30 mile radius of their corresponding test centre. Eligible volunteers were required to obtain consent from their General Practitioner to take part in the study and any volunteer was excluded if they had any of the following: prior history of coronary heart disease, known type 1 or 2 diabetes, liver or respiratory failure, gout, taking lipid lowering or anti-hypertensive drugs, history of obesity with known cause (ie Cushing's disease, hypothyroidism), previous gastric or weight-loss surgery, taking any weight loss drug (including Orlistat or Sibutramine), clinical depression, eating disorders, drug or alcohol abuse, any malabsorptive state (including lactose intolerance), treatment for a malignancy, pregnancy or breastfeeding.

Screening procedures resulted in 293 (79 males, 214 females) subjects being screened as eligible for the study. Due to the disproportionate number of female volunteers (70%), subjects were stratified by gender and then randomised across each site to the four diets (Atkins n = 57, WW n = 58, SF n = 59, RC n = 58) and a delayed treatment control group (n = 61). In this way, subjects attending the group based programmes were not located together and furthermore regional food intake differences would be accounted for by a geographically representative spread of participants around the UK. All centres started the study within a 6 week period thus enabling seasonal variation in food availability to be minimised.

### Assessment instruments

At baseline (prior to randomisation), and at two months, subjects were asked to complete a 7-day food (with estimated weights) and concurrent with a 7-day activity diary [12]. The activity diary enabled subjects to record what they were doing minute by minute across a 24 hour period, and provided with an extensive list of activities from which to allocate their time. Subjects were instructed how to complete the food diary using estimated weights of foods and beverages from a comprehensive list supplemented by photographs; telephone support was available if required. When subjects returned to the test centres for anthropometric measurements, they were individually de-briefed as they returned their diary and any queries regarding food or drink consumption or activities were resolved. All diaries were analysed centrally at the University of Surrey. Nutrient intake was assessed using WinDiets (Research Version, the Robert Gordons University) by Registered Dietitians or supervised students. Micronutrient intakes were compared to current UK reference nutrient intake (RNI) values [13].

In addition to the nutrient analysis, the number of fruit (including fruit juice) and vegetable portions were counted assuming a standard portion size of 80 g. Totals per week were then divided by 7 to provide an average daily intake of fruit and vegetables.

### Validation of energy intake

Activity diaries were coded into minutes per day of time spent in sleeping, light, moderate or vigorous activity. Minutes in each category per day were multiplied by a metabolic equivalent (MET) value to give a total daily MET value using the following values obtained from the Compendium of Physical Activity [14,15]: sleeping (MET 1), light (MET > 1.5 < 3.5), moderate (MET 4 – 6), vigorous activity (MET > 6). The totals for daily MET values were then summed to give a MET score for the week and then divided by 24 hours to give a physical activity level (PAL). Each subject was assigned a physical activity level based on their PAL score. The mean PAL value for the group at baseline was 1.27 (SEM 0.004). Total energy expenditure was predicted (pTEE) using the Institute of Medicine of the National Academies for overweight and obese adults formulae[16] which utilises age, height, weight, gender and physical activity based on PAL score. A ratio of reported energy intake (rEI) to pTEE (rEI:pTEE) was calculated for each subject at baseline.

The procedure for identifying mis-reporters of energy intake at baseline followed the method of McCrory et al. [17] where those reporting energy intakes plus or minus 1 SD for the agreement between rEI and pTEE are regarded as physiological implausible. This method takes into account the within subject errors associated with each parameter and is based on the principles of the agreements between PAL and rEI and BMR, originally outlined by Black[18]. These include a value of 8.2% being the coefficient of variation of the technical error for measuring TEE using doubly labelled water (CV_tmTEE_) and 17.7% for measurement errors in predicted TEE (CV_wpTEE_). The method can be mathematically described as follows:

$$\begin{array}{c} \text{ISD}=\surd ({\text{CV}}_{\text{wEI}}^{2}/\text{d})+{\text{CV}}_{\text{wTEE}}^{2}\\ =\surd ({\text{CV}}_{\text{WEI}}^{2}/\text{d})+{\text{CV}}_{\text{wpTEE}}^{2}+{\text{CV}}_{\text{tmTEE}}^{2} \end{array}$$

CV_wEI _= within subject coefficient of variation in energy intake

d = number of days of food records

Using these equations physiologically implausible energy intake reporters at baseline were identified by excluding rEI +/- 23% (i.e 1 SD). The records not excluded then formed a 'plausible reporters' sub-group which was defined by those who had reported energy accurately at baseline making the recognised assumption that they would continue to record food intake accurately at 2 months [19]. The plausible reporter sub-group were only used to examine changes in energy intake over the 2 month period. When analysing the micronutrient content all dietary records were utilised (ie. plausible and implausible) as under reporting would, if anything, under estimate micronutrient intake.

### Provision of dietary programmes

Subjects randomised to the group based programmes (RC and WW) attended their most locally based group due to the wide geographical spread of participants. The cost of attending the group classes were refunded on provision of a receipt. Subjects randomised to the Atkins diet were provided with a copy of the book but no further advice was given [4]. Subjects allocated to Slimfast were provided with the Slimfast Support pack and were given a one week supply of meal replacement shakes (2 meal replacements per day). After that time, the cost of up to 2 meal replacements per day were refunded on the provision of receipts. The control group were asked not to alter their current diet or exercise levels and were offered the diet of their choice free of charge for 6 months after the study was completed. The cost of travelling to test centres was refunded to all participants. Subjects were not given any individual dietary counselling by the study staff. No attempt was made to standardise energy intake across groups as the overall purpose of the study was to determine the relative effectiveness of commercial diet programmes in overweight, but otherwise healthy subjects, who were free to interpret the dietary regimen as they chose.

### Statistical analysis

Summary statistics are presented as mean and standard error. Differences between groups at baseline for continuous outcomes were compared using Student's t-tests, except where the Shapiro-Wilk test indicated non-normal distribution of data, when the non-parametric Mann Whitney U test was used. For categorical variables, the chi-square test was used to investigate between group differences. For normally distributed data where homogeneity of variance was confirmed, ANOVA was used to explore between group differences and where the overall result provided evidence of significant group differences, post hoc comparisons were conducted. For the data that was not normally distributed the non-parametric Kruskal-Wallis test was used. Changes over time for each diet group (baseline to two months) were explored using generalizing estimating equations (GEEs) with an identity link function and an exchangeable correlation structure, thus we were able to adjust for the correlation between repeated measurements on the same participant. Robust variance estimate techniques were used to calculate standard errors and confidence intervals. All p-values were two-sided. To account for multiple comparisons a p-value of less than 0.01 was considered statistically significant. Data were analyzed using Stata version 10.0 (StataCorp College Station, TX, USA).

## Results

After randomisation to diet group, the average age of subjects participating in the study was 40.3 years (sd 10.2, range 20–61 years), mean BMI was 31.7 kg/m2 (sd 2.7, range 27 – 38), mean waist circumference was 101.4 cm (sd 10.4, range 80 – 128 cm). There were no diet group or centre differences in these baseline characteristics. Smoking was reported in 17% of women and 10% of men.

**Figure 1:**
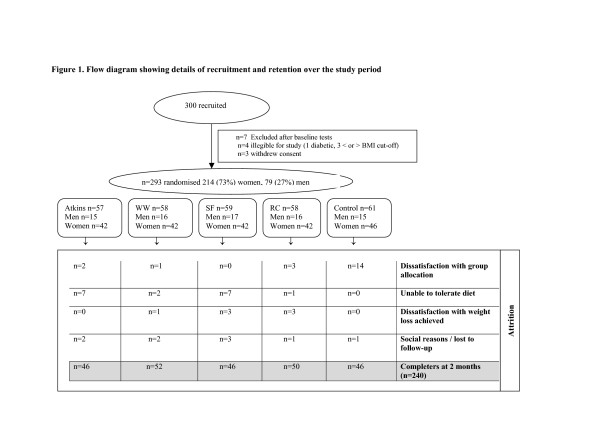
Participant Flow.

The overall attrition rate after two months was 18% (n = 53), with no significant difference between centres. The main attrition occurred immediately after randomisation primarily in the control group with 23% withdrawing because they did not wish to delay a weight loss attempt (see Participant Flow Figure 1).

Seventy six percent (n = 223) returned 7-day completed diet and activity diaries at baseline. After two months, 172/234 participants returned diaries (74%) with no significant difference in the return of diaries by group or centre.

### Diet composition prior to randomisation to diet group and mis-reporting

Reported energy intakes are presented in Table 1 for the whole cohort and for the plausible reporters sub-group (defined as %rEI:TEE +/- 1 SD). There were no significant differences at baseline between groups for reported energy intake. Mis-reporting of energy intake at baseline was common, with subjects tending to under-report food intake when compared with estimated energy requirements (mean rEI:TEE was 81.1% sem 1.5, range 22–137%). 60% subjects were deemed to have provided 'plausible' dietary records at baseline, with no significant differences between diet groups or between genders (37% men and 41% women mis-reported). There were no differences in either rEI at baseline or rEI/kg body weight between the diet groups for the whole cohort or for the 'plausible reporters' group.

**Table 1:** Average reported energy intake (mean and standard error of mean) at baseline and after two months after allocation to diet group (see appendix for explanation of statistics)

	Atkins		WW		SF		RC		Control	
	Basal	2 m	Basal	2 m	Basal	2 m	Basal	2 m	Basal	2 m
n	44	31	53	45	44	36	45	31	37	29
^a^Energy (kJ/day)										
Mean	9550	6809	9706	6084	9512	6076	10149	6417	9512	7947
Sem	404	415	427	239	456	316	409	201	367	486

^b^Energy kJ/kg/day										
Mean	105	80	108	74	106	70	114	81	100	97
Sem	4.5	4.7	3.9	2.6	3.9	3.7	29.2	2.6	3.6	6.5

^c^Plausible reporters only										
Energy kJ/kg/day	n = 17	n = 15	n = 23	n = 23	n = 15	n = 13	n = 20	n = 18	n = 12	n = 8
Mean	121	86	123	80	119	78	121	81	115	117
Sem	3.8	6.1	2.8	3.9	5.7	8.5	3.8	3.2	3.4	17

### Macro and micro-nutrient composition of diet at baseline

At baseline the average % of energy from macronutrients comprised of 42% carbohydrate, 37% fat, 16% protein, 5% alcohol for the whole cohort. In terms of fat content, the average baseline diet contained 30.8 g saturated fat, 14.8 g polyunsaturated and 28.4 g monounsaturated fat. All micronutrient intakes exceeded their respective RNI values with the exception of potassium where the average intake was 95% RNI. There were no significant differences between the baseline diets in terms of macro, micro-nutrient or % energy derived from alcohol. Therefore, all data presented are changes for each diet group from baseline measurement.

### Macronutrient changes

Alteration of energy intake across all diet groups was apparent over the study period with significant falls in total energy intake and energy intake/kg/body weight recorded (Table 1). This was mirrored by analysis of the 'plausible reporters' sub-group which showed a significant reduction in energy intake (kJ/kg/day) between baseline and two months (F_4,72 _= 3.85, p = 0.007), with significant differences between control and all active diet groups. These falls in energy intake are substantiated by the physiological measures of weight loss. Although changes in body weight are fully reported elsewhere [11], in summary, the mean (sd) weight losses (kg) over the 2 month study were as follows: Atkins 5.2 (4.4), WW 4.7 (3.2), SF 3.7 (3.5), RC 4.0 (3.3), Control 0.4 (1.8), with all diet groups being significantly greater than control but there were no differences between active diet groups.

**Figure 2:**
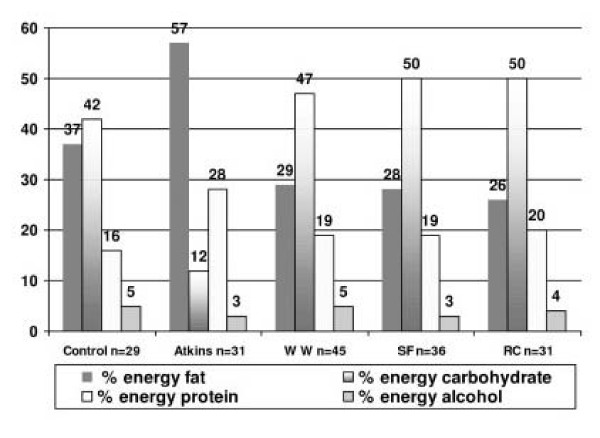
Alteration in percentage of energy from macronutrients from baseline and after 8 weeks of dieting.

The patterns of change in macronutrient composition were in line with the expected changes if subjects were choosing foods consistent with the recommendations of each dietary regimen indicating dietary compliance and was completed using all available data. See Figure 2.

In the Atkins dieters, there were highly significant shifts (p < 0.001) in the percentage (%) energy derived from all macronutrients from baseline to 2 months, as follows: 11% increase in protein energy (95% C.I. -13.7, -9.1); 10% increase in energy from fat (95% C.I. -24.1, -16.3); 29% fall in CHO energy (95% C.I. 24.7, 32.6) and a 3% fall in energy derived from alcohol (95% C.I. 1.28, 4.76). Overall, there was a fall of 30% in mean total energy intake (95% C.I. 381, 900).

The RC diet recommends participants chose foods with less than a 4% fat content. Significant dietary shifts did occur with % energy from fat falling on average by 11% (95% C.I. 7.98, 13.2). In contrast, protein energy increased by 4% (95% C.I. -5.86, -2.9), CHO rose by 8% (95% C.I. -9.83, -6.19). There was no significant change in % energy derived from alcohol. Overall there was a fall of 37% in mean daily energy intake (95% C.I. 680, 1102).

The WW diet recommends a low fat intake, and this was reflected in a significant 7% reduction in dietary fat (95% C.I. 5.7, 9.79) between baseline and two months. CHO energy rose significantly by 4% (95% C.I. -6.07, -2.55) to provide 47% energy; dietary protein followed a similar pattern with a significant increase from baseline to 2 months (95% C.I. -4.78, -2.52). There were no significant changes in % energy derived from alcohol. Overall there was a fall of 38% in mean daily energy intake (95% C.I. 700, 1100).

The use of meal replacements resulted in the SF group having a significantly lower fat (% energy) diet at 2 months (95% C.I. 4.34, 9.93), with corresponding significant increases in CHO energy by 7% (95% C.I. -10.99, -3.96) and in protein energy which provided 19% of total energy intake at 2 months (95% C.I. -8.6, -4.78). Alcohol consumption also declined from baseline to two months by 2% energy (95% C.I. 1.01, 3.61). Overall there was a mean fall of 37% in daily energy intake (95% C.I. 627, 1151).

In the control group, there was a non-significant (10%) fall in reported energy intake from baseline to two months (p = 0.06), with no significant alterations in % energy derived from fat, protein, CHO or alcohol.

### Alteration in fat intake

**Figure 3:**
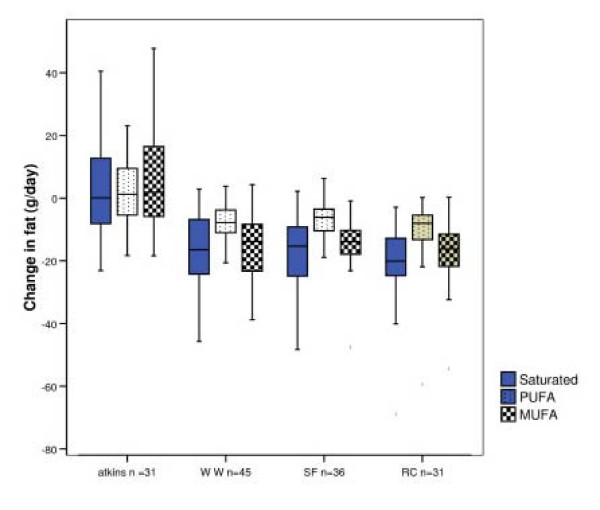
Change in fat (g/day) between baseline and 2 months for those following the Atkins, Weight Watchers, Rosemary Conley and Slimfast diets.

Of interest in this analysis, is the effect of the different dietary programmes on the type and amount of fat intake and in particular differences between the low CHO and low fat diets (WW and RC). Change in dietary fat intake was calculated from baseline to 8 weeks in terms of absolute intake (g/day), see Figure 3. This shows that although there was a proportional increase in % energy from fat in the Atkins dieters, there were no significant changes in absolute intake of fat per day or in the quantity of saturated fat consumed. It should be noted, the amount of saturated fat consumed fell significantly on all the other diets and also in the control group.

### Changes in micronutrients

Micronutrient intakes reported as a proportion of recommended daily intakes (RNI) are summarised in Table 2 using data for completers only. All of the Atkins group were calculated without micronutrient supplementation and the RNI for thiamin was calculated as 0.4 mg/4200 kj reported energy intake. At baseline, the only micronutrient consistently below RNI was potassium, this showed a trend to drop further away from the RNI in all diet groups over time. Changes within diet groups are summarised as follows:

**Table 2:** Micronutrient intake (mean % RNI) and percentage change in RNI over the  2 month study period by diet group for individuals with data recorded at baseline and at follow-up

	Atkins		Wt Watchers		Slimfast		R Conley		Control	
	Baseline	2 m	Baseline	2 m	Baseline	2 m	Baseline	2 m	Baseline	2 m
n	30		45		34		30		26	
Vitamin A										
Mean	182	175	128	104	119	203	158	105	136	118
SEM	35	47	10	9	11	58	21	14	17	13
%difference (95%CI)	4 (-67,75)		-23 (-84,39)		80 (12,148)		-54 (-125,17)		-49 (-125,26)	
P-value	0.91		0.47		0.02		0.14		0.19	
Thiamin										
Mean	223	146	240	323	198	250	235	449	274	178
SEM	34	9	40	91	15	16	37	250	65	15
%difference (95%CI)	-96 (-283,90)		98 (-68,264)		37 (-141,215)		208 (14,402)		-70 (-267,128)	
P-value	0.31		0.25		0.68		0.40		0.49	
Riboflavin										
Mean	158	138	161	128	147	167	169	151	143	134
SEM	9	11	7	7	7	12	11	8	7	8
%difference (95%CI)	-19 (-36,-3)		-30 (-44,-16)		18 (3,34)		-14 (-30,3)		-9 (-27,8)	
P-value	0.02		<0.00		0.02		0.10		0.29	
Niacin										
Mean	310	353	314	253	301	257	321	284	307	283
SEM	14	29	12	10	12	14	13	11	20	18
%difference (95%CI)	44 (8,79)		-55 (-96,-25)		-52 (-86,-18)		-33 (-69,3)		-18 (-56,19)	
P-value	0.02		<0.001		0.003		0.07		0.34	
Folate										
Mean	139	93	132	116	125	133	138	133	126	114
SEM	9	8	8	5	7	7	10	9	8	7
%difference (95%CI)	-42 (-57,-26)		-13 (-26,0)		6 (-9,21)		-2 (-18,14)		-8 (-25,8)	
P-value	<0.00		0.05		0.41		0.998		0.34	
Vitamin C										
Mean	224	132	242	224	248	326	309	379	275	205
SEM	23	19	21	21	29	69	39	57	40	42
%difference (95%CI)	-89 (-178,19)		-13 (-90,64)		65 (-21,151)		107 (17,197)		-78 (-174,19)	
P-value	0.06		0.74		0.14		0.02		0.11	
Calcium										
Mean	125	92	130	87	133	139	138	113	123	111
SEM	8	7	6	4	11	6	10	15	10	8
%difference (95%CI)	-33 (-51,-11)		-39 (-58,-20)		2 (-19,24)		-22 (-44,1)		-22 (-46,1)	
P-value	0.004		<0.00		0.82		0.06		0.06	
Magnesium										
Mean	106	75	112	84	106	104	115	97	102	95
SEM	6	7	5	3	4	4	4	4	4	6
%difference (95%CI)	-31 (-41,-21)		-25 (-34,-17)		-3 (-12,7)		-16 (-26,-6)		-5 (-15,6)	
P-value	<0.001		<0.001		0.56		0.002		0.38	
potassium										
Mean	96	67	98	77	95	88	104	88	93	84
SEM	5	4	5	4	4	4	4	4	4	4
%difference (95%CI)	-28 (-37,-19)		-19 (-27,-12)		-8 (-17,0)		-12 (-21,-3)		-8 (-17,2)	
P-value	<0.001		<0.001		0.05		0.007		0.11	
iron										
Mean	136	91	121	97	111	130	126	110	116	104
SEM	11	9	10	9	8	9	9	8	11	9
%difference (95%CI)	-43 (-59,-28)		-24 (-37,-11)		17 (2,31)		-16 (-31,0)		-11 (-27,5)	
P-value	<0.001		<0.001		0.03		0.05		0.18	
zinc										
Mean	119	139	113	88	117	196	123	96	118	103
SEM	6	9	4	4	5	18	5	4	8	6
%difference (95%CI)	15 (-4,35)		-24 (-41,-7)		74 (55,92)		-26 (-45,-6)		-12 (-33,9)	
P-value	0.13		0.005		<0.001		0.01		0.25	
selenium										
Mean	100	149	116	86	97	97	103	79	93	87
SEM	6	41	13	7	5	4	5	5	7	12

%difference (95%CI)	49 (18,79)		-25 (-51,1)		-2 (-31,27)		-27 (-57,4)		-6 (-39,26)	
P-value	0.002		0.06		0.89		0.119		0.70	

#### Atkins group

There were significant falls in %RNI for folate, magnesium, calcium, iron and potassium and a significant increase in selenium.

#### Weight Watchers group

There were significant declines in %RNI for riboflavin, niacin, potassium, calcium, magnesium, iron and zinc.

#### Slimfast group

There was a significant decline for niacin and a rise in %RNI for zinc after 2 months.

#### Rosemary Conley group

There were significant decline in %RNI for magnesium, potassium and zinc.

#### Control group

There were no significant alterations in micronutrient %RNI in the control group.

### Iron intake

Iron is the major micronutrient at risk of deficiency in the UK diet today. An examination of iron intake for those undertaking weight reducing diets is worthwhile as it is often this mineral that is lacking in the diets of women of child-bearing age. In this study, there were significant differences in iron intake between males and females at baseline. At baseline, men had an absolute iron intake (median 14.6 mg, range 5.7 – 27.1 mg/day) that was significantly higher (p < 0.001) than women (median 11.9 mg range 5.6 – 23 mg/day). Changes in intake of iron (mg/day) from baseline to two months demonstrated a significant effect of diet group for women only (Chi Sq 7.934, p = 0.94 for men, chi Sq 21.2 p < 0.001 for women).

### Dietary fibre and fruit and vegetable intake

Intake of dietary fibre was on average 17.7 g/day (sem 0.39); with no diet group differences but gender differences were apparent with men consuming on average 3 g/day more dietary fibre than women (95% C.I 1.13, 5.03). Baseline intake of non-starch polysaccharide (NSP) was below the recommended intake of 18 g/day (mean intake 12.8 g, sem 0.27).

The median number of fruit and vegetable portions consumed at baseline was 17 portions (IQR 16.25) per week (2.4 portions per day). Only 12% of the entire cohort achieved the UK recommended intake of ≥ 5 fruit and vegetables portions per day at baseline. There were no between diet group differences in portions of fruit and vegetables eaten at baseline.

Only the WW diet led to a significant increase (Z -3.21, p = 0.001) of fruit and vegetables and this amounted to less than one portion per day (0.79 portions per day). There was a trend towards an increase in the RC group (0.53 portion increase, p = 0.06); there were no significant shifts for the other diets tested. Although portions of fruit and vegetables eaten on the Atkins diet did not alter over time, there was a significant reduction in NSP intake in the Atkins dieters. A repeated measures ANOVA of NSP intake from baseline to 2 months of all diet groups showed a significant effect of diet group (F = 8.43, p = <0.001) and post hoc testing demonstrated a significant reduction in NSP (12.8 g at baseline to 5.1 g at 2 months) for the Atkins group compared to all the other diets.

## Discussion

The analyses presented in this paper demonstrate that free living subjects are able to make significant dietary change in line with instructions provided by commercial companies regardless of whether this information is given at group classes or as written instructions. Substantial alteration in participants macro-nutrient composition were recorded and these are supported by the recorded weight losses obtained during the 'Diet Trials' study; indicating compliance with the allocated dietary regimen [11].

In the low CHO Atkins diet, we have shown that subjects were able to reduce their CHO intake substantially without the need for individualised dietary counselling. Furthermore, the energy deficit induced can be attributed to a reduction in overall energy intake with non-replacement of CHO energy and no substantial increase, in absolute terms, of dietary fat. The low fat diets, WW and RC both led to reductions in saturated fat intake, as both proportions of energy and in absolute terms, but it is of interest that the Atkins dieters in this study did not substantially increase their absolute intake of saturated fat which is what might have been anticipated. The effects of these diets on lipid profile are reported elsewhere [20] but confirm that there was no substantial adverse effect of the Atkins diet on lipid profile. This is in addition to the general benefit of reducing cardiovascular risk factors by weight body loss per se in obese adults regardless of the macronutrient diet composition [21-24].

Baseline intake of fruit and vegetables were lower than the recommended '5 a day' that is encouraged in the UK but was similar to that reported as 'usual' intake for adults in the UK [25]. Although it is not surprising that those following a low CHO approach would not increase their fruit and vegetable intake over time, it is interesting that after two months of this diet, most people had not decreased their 'usual' level of fruit and vegetables. This is particularly notable given the timing of the data collection in this study. The Atkins diet only recommends a very low CHO intake (5–10 g/day) for the first few weeks of this approach so after two months, subjects may have chosen to use their CHO allowance for fruit and vegetables instead of bread and cereals. This is supported by the micronutrient profile of the Atkins dieters, who tended to have a reduction in iron and niacin, probably due to a fall in the intake of cereal and flour, which is fortified in the UK, on the low CHO diet. The significant reduction in the Atkins dieters of NSP and the generally low intake of dietary fibre overall may have implications for bowel health in the longer term.

All the other commercial diets encourage an increase in fruit and vegetables partly to increase the satiety of the meals and also to replace high fat, high sugar snacks. A significant increase in fruit and vegetables was only observed in those following the WW diet but this increase was less than one portion per day. These disappointing findings suggest that people remain resistant to the advice to 'eat more fruit and vegetables' even when they are advised to as part of a modified weight loss programme.

On the whole, micronutrient intake remained above the RNI for most nutrients on all the commercial diets even with the degree of mis-reporting of energy intake. Bearing in mind the degree of under-reporting established in this study, there is little evidence to suggest that subjects following self-selected weight reducing diets in the long term would be at risk of micronutrient deficiency. Some subjects following the Atkins diet may have been following advice and taking a daily multi-vitamin supplement which is recommended in the book but this was not analysed. Gender differences were apparent, with women tending to reduce their daily iron intake with energy restriction. Those with high iron requirements due to menstrual losses may be at risk of iron deficiency if they were dieting for long periods of time. This could occur on low either of the low fat approach and the low CHO diet. Meal replacement products that ensure adequate micronutrient provision appeared to offer an advantage in this respect.

This analysis aimed to compare the nutritional composition of a low CHO diet to low fat diets. We find little evidence of short-term detrimental effects on nutrient intake with a low CHO approach compared to a low fat approach. Folate intake was only just above recommended levels on all the diets tested, although it fell on the Atkins diet at 2 months but still met 93% of the RNI. Women planning a pregnancy would be well advised to take additional folate while following any of these weight reducing regimens. Health professionals should be aware that in the UK, fortified breakfast cereals and bread flour contribute substantially to iron and B complex vitamin intakes and when these foods are restricted, other sources of these nutrients need to be found. The assumption that low CHO diets become very high in protein due to increased consumption of meat is not substantiated by these data.

There is little published data on nutrient adequacy in those trying to lose weight. Ashley et al (2007) [26] reported nutrient profiles of two groups one following a meal replacement approach and the other more traditional structured low fat diet approach. Despite these groups receiving supervision by a dietitian, the traditional diet had lower intake of calcium and other minerals compared to the meal replacement group, leaving the authors to suggest some benefits of taking fortified food/drink while following an energy restricted diet.

This study provides some information on the usual diet of overweight people in a reasonably large sample of free living subjects from geographically diverse areas of the UK. There are a number of limitations with the methodology used that need to be considered when interpreting the results. First, nutrient intake profiles are the result of self-reported measures of diet which have a number of known limitations, the most significant of which has to be the well documented and long standing issue of mis-reporting of food intake which is common in overweight and obese populations [19,27-29]. It was beyond the scope of this study to validate the energy expenditure estimations using the gold standard of doubly labelled water. The challenge of collection of accurate food intake data has directed the analysis presented in this paper to focus on change in nutrient profile from baseline measures rather than rely only on absolute nutrient values. When describing the relative adequacy of the diet in terms of %RNI of micronutrients, the effect of under-reporting food eaten would be likely to lead to under estimates of actual intake. We have been able to use estimated energy expenditure values to derive individual energy requirements and utilise this information to calculate cut-offs for mis-reporting rather than rely on the blunter instrument of the Goldlberg cut-offs for under reporting of energy intake [30]. Food recording for only 7 days may also under represent some micronutrients as longer recording periods would be preferable to ascertain habitual intake. Additionally, not all subjects provided food records so combined with the attrition rate, we report on a small sample size. However, support that the nutrient profiles obtained in this study are representative comes from the general agreement with the nutrient profile of the UK population obtained from National Dietary Surveillance surveys [31]. The decline of energy intake described on a per kg body weight basis in the 'plausible reporters' group concurs with the actual weight loss achieved in this study and supports the relative direction of accuracy of the food reporting procedures overall. Thus, regardless of dietary energy macronutrient composition, weight loss will occur on popular diets if an overall energy deficit is achieved. The final limitation is connected to the development of the RNIs themselves and their applicability at an individual level. Some nutrients such as selenium and calcium, these may actually prove to be a lot higher for 'optimal health' as opposed to their current use which are set at a population level to prevent deficiency states.

To conclude, this is a novel study which provides comprehensive dietary data on a substantial cohort of subjects following four popular diets without supervision. Health professionals generally would consider three of the diets as nutritionally acceptable (WW, RC and SF) and one diet (Atkins) being controversial. Comparisons of pre- and post-intake indicated dietary compliance. Baseline data suggested overall nutritional adequacy and none of the diets resulted in micronutrient insufficiency or an increase in absolute fat intake which has been a common criticism of low CHO diets. An inadequate intake of dietary fibre was noted in this diet. The caveats to this study are the inherent errors of dietary assessment and that findings may not be generalisable to certain subgroups within the population with specific nutritional requirements, particularly women with raised iron requirements and those with increased calcium needs. This analysis provides reassuring and important evidence for the effectiveness and nutritional adequacy of four commercial diets in weight management for the general public which are particularly pertinent for community and public health nutritionists and those working in primary care. It is suggested that commercial companies work in partnership with health professionals to identify and intervene with high risk clients, such as those planning pregnancies, to provide more individualised dietary advice.

## Competing interests

KRF receives consulting fees for serving on the scientific advisory panel of Slimming World, a company that offers a support service for weight loss. This company was not involved in this trial but as it is similar to Rosemary Conley and Weight Watchers, the conclusions may have implications for the company.

## Authors' contributions

All authors were involved with data collection. RH, AH, MS were in particular involved in the diet and nutrient analysis. HT and MBEL drafted the manuscript with RW completing the statistical analysis, all authors have been involved in reviewing the manuscript.

## Appendix

### Statistical analysis and footnotes for Table 1

ANOVA a: 2 m data: F_4,172 _= 5.1, p < 0.001, post hoc differences between control and SF, control and WW, control and RC;

ANOVA b. 2 m data: F_4,172 _= 5.5, p < 0.001, post hoc differences between control and SF and control and WW;

ANOVA c: 2 m data: F_4,77 _= 3.85, p = 0.007, post hoc differences between control and all other groups
